# How human T-cell leukemia virus type 1 induces diseases

**DOI:** 10.1186/1742-4690-10-S1-O15

**Published:** 2013-09-19

**Authors:** Masao Matsuoka

**Affiliations:** 1 Institute for Virus Research, Kyoto University, Japan

## 

Human T-cell leukemia virus type1 (HTLV-1) is the causal agent of adult T-cell leukemia (ATL), and inflammatory diseases, such as HTLV-1 associated myelopathy (HAM), uveitis, alveolitis and infective dermatitis. Approximately, 5% of carrier will develop ATL after a long latent period. A characteristic of this virus is that HTLV-1 can transmit only by cell-to-cell contact. Therefore, HTLV-1 increases infected cells *in vivo* to facilitate its transmission.

Previous studies suggest that Tax plays central roles in leukemogenesis by HTLV-1. However, Tax expression is frequently impaired in ATL cells. The *HTLV-1 bZIP factor* (*HBZ*) gene is transcribed from minus strand of provirus using 3’ long terminal repeat (LTR) as the promoter/ enhancer. Among ATL cases, only the *HBZ* gene is expressed in all ATL cases, suggesting an importance of the *HBZ* gene in ATL cells. Indeed, transgenic expression of the *HBZ* gene induced T-cell lymphoma and inflammatory diseases. Furthermore, regulatory T (Treg) cells increased in HBZ-transgenic (HBZ-Tg) mice. As a mechanism, HBZ induces transcription of the *Foxp3* gene through enhanced Smad/ TGF-β pathway and converts infected cells to Treg cells. Concurrently, HBZ impairs function of Treg via interaction of HBZ with Foxp3 and associated factors. Thus, HBZ increases functionally impaired Treg cells *in vivo*, which resembles to phenotype of HTLV-1 infected cells and ATL cells. It is speculated that HTLV-1 infected Treg cells escape the host immune surveillance by acquired phenotype of Treg cells. Treg cells derived from HBZ-Tg mice progressively lost Foxp3 expression *in vitro* and produced IFN-γ, indicating that HBZ induces inflammation through unstable Foxp3 expression. Thus, HBZ is responsible for both T-cell lymphoma and inflammatory diseases.

HBZ has pleiotropic functions as well as Tax. HBZ enhances TGF-β/Smad pathway while Tax inhibits it. Likewise, HBZ suppresses canonical NF-κB whereas Tax activates canonical and noncanonical NF-κB pathways. Thus, HBZ has opposite effects to Tax in many pathways. Interplay between HBZ and Tax likely coordinates to promote viral replication and proliferation of infected cells, leading to important roles in the pathogenesis.

